# Immunotherapy Treatments of Warm Autoimmune Hemolytic Anemia

**DOI:** 10.1155/2013/561852

**Published:** 2013-09-11

**Authors:** Bainan Liu, Wangang Gu

**Affiliations:** Department of Immunology, Zunyi Medical College, Zunyi, Guizhou 563003, China

## Abstract

Warm autoimmune hemolytic anemia (WAIHA) is one of four clinical types of autoimmune hemolytic anemia (AIHA), with the characteristics of autoantibodies maximally active at body temperature. It produces a variable anemia—sometimes mild and sometimes severe. With respect to the absence or presence of an underlying condition, WAIHA is either idiopathic (primary) or secondary, which determines the treatment strategies in practice. Conventional treatments include immune suppression with corticosteroids and, in some cases, splenectomy. In recent years, the number of clinical studies with monoclonal antibodies and immunosuppressants in the treatment of WAIHA increased as the knowledge of autoimmunity mechanisms extended. This thread of developing new tools of treating WAIHA is well exemplified with the success in using anti-CD20 monoclonal antibody, Rituximab. Following this success, other treatment methods based on the immune mechanisms of WAIHA have emerged. We reviewed these newly developed immunotherapy treatments here in order to provide the clinicians with more options in selecting the best therapy for patients with WAIHA, hoping to stimulate researchers to find more novel immunotherapy strategies.

## 1. Introduction

Warm autoimmune hemolytic anemia (WAIHA) is characterized by the autoantibodies directing against patient's own antigens on red blood cells with the best reactive temperature at 37°C and accounts for about 50% to 70% of all cases of autoimmune hemolytic anemia [1, 2]. It can be classified by the presence or absence of pathophysiologically or etiologically associated underlying diseases. When no recognizable underlying disease is present, the WAIHA is termed primary or idiopathic. When the WAIHA appears to be a manifestation or complication of an underlying disorder, it is termed secondary. The secondary conditions may be primary immunodeficiencies such as common variable immunodeficiency, hematologic malignancies, infections, tumors, or drugs [3]. Using strict classification criteria, primary WAIHA comprises about 50 percent of cases. Chronic lymphocytic leukemia (CLL) and lymphomas account for about half of secondary WAIHA cases. Autoimmune diseases, particularly systemic lupus erythematosus (SLE), account for a considerable proportion of the remaining secondary WAIHA cases [1]. The diagnosis of this rare disease is usually made on the laboratory findings, mainly on the direct antiglobulin test (DAT). Clinicians should carefully make the treatment decisions based on the diagnostic information and should know what type of the antibody is involved and if the disease is primary or secondary [4]. WAIHA can be caused by a number of different classes of antibody, with IgG, and IgM antibodies being the main causative classes. IgA class antibodies are present in approximately 14% of patients with WAIHA and are almost always associated with IgG or IgM [5, 6]. If no underlying causes can be identified in WAIHA, it is truly primary. Treatments are variable by the different pathologic conditions accordingly. 

Recently, advances in elucidating the mechanisms of autoimmunity have been made. Attributing to this newly extended knowledge of autoimmunity, novel approaches to treating autoimmune diseases are also being worked out. For patients with WAIHA, current recommended therapeutic strategies include glucocorticoids as the first-line treatment and splenectomy for patients who do not show a satisfactory response to glucocorticoids. Intravenous immunoglobulin and alemtuzumab, as well as other immunosuppressive drugs, have also been successfully used in patients with WAIHA refractory to glucocorticoids and splenectomy [7]. The progress which is recently made is the use of rituximab shown to be effective in treating WAIHA patients, providing a new thread of treating WAIHA by immunotherapy. Treatments of WAIHA have been reviewed by several investigators [8–11], but none of them focused on the immunotherapy of this uncommon, severe, sometimes life-threatening disease.

Immunotherapy is defined as “the treatment of disease by inducing, enhancing, or suppressing an immune response”. It is classified as activation immunotherapy if designed to elicit or amplify an immune response and classified as suppression immunotherapy if the immunotherapy reduces or suppresses the immune response. In consideration of the emphasis of immunotherapy of autoimmunity-related WAIHA on suppressing the immune reactivity, this review centers mainly on the immunosuppression of WAIHA through the recent advances in immunotherapy.

## 2. Immunotherapy of WAIHA with Immunosuppressants

Immunosuppressants dampen the immune response or restore balance among immune system components. They are primarily used to prevent or treat disease flares in autoimmune diseases and to prevent allograft rejection after organ transplantation. As an uncommon autoimmune disease, the mainstay of therapy of WAIHA is immunosuppression with corticosteroids. Allgood and Chaplin followed 43 patients, of whom 32 (74%) responded to corticosteroids but 25 (78%) of the responders relapsed in the 9 months to 11 years of follow-up [12]. Zupanska et al. demonstrated similar results with the majority of patients initially responding, but only 19 (46%) of 41 continue to respond to treatment after 3 weeks [13]. This means around 80% of patients respond promptly to corticosteroids; however, a proportion of responders will have disease relapse after the steroid-induced remission. Petz stated that about 50% of corticosteroids responders require maintenance therapy [14]. For patients who do not respond to corticosteroids, other immunosuppressants, including cyclosporin and azathioprine can then be used [15]. An example of application of cyclosporin is documented by Pogglitsch et al. [16]. They successfully treated a 60-year-old patient suffering from an idiopathic warm autoimmune hemolytic anemia by cyclosporin A after the therapy of prednisone. Worlledge et al. reported 4 corticosteroid-treated patients who received azathioprine and demonstrated that anemia was controlled in 1 patient [17].

The above data indicate that the immunotherapy of WAIHA with immunosuppressants has always been used and will be continued in clinics before better treatments emerge. Especially for first-line therapy, steroids will remain the preferred treatment. However, most of the immunosuppressive agents that have been tried in WAIHA patients often cause various side effects, sometimes induce refractoriness to the treatment, or lead to a relapse condition. New treatment options that can reduce the side effects or improve the benefit/risk ratio should be developed in urgency. Recently, this is getting closer to success as knowledge of WAIHA is rapidly increasing, especially on pathogenesis, associated disorders, and aspects of transfusion immunology.

## 3. Immunotherapy of WAIHA with Monoclonal Antibodies (mAb)

### 3.1. Treatment by Rituximab

Rituximab is a humanized monoclonal antibody directed against CD20 on pre-B cells and mature B lymphocytes. Binding of rituximab to CD20-positive cells results in cell death via a combination of antibody-dependent cell cytotoxicity, complement activation, and apoptosis [18, 19]. Due to these properties, it has been used to deplete B lymphocytes and reduce autoantibody production in the treatment of autoimmune disorders mediated by autoantibodies including the treatment of relapsed or refractory warm autoimmune hemolytic anemia patients. Its treatment efficacy for both children and adults with WAIHA has been well documented. Quartier et al. used rituximab to treat 6 children with refractory WAIHA and achieved a 100% complete response rate [20]. Zecca et al. treated 13 refractory WAIHA children: a complete response (CR) was achieved in 11, while two failed to respond [21]. In the adult setting, D'Arena et al. treated 11 patients with refractory primary WAIHA by rituximab in a standard dose of 375 mg/m^2^ in a retrospective study [22]. Eight patients achieved a complete response (CR), and 3 of them achieved a partial response (PR), but 6 patients still had discrete laboratory signs of hemolysis. All patients remained in either CR or PR, at a mean follow-up of 604 days. The longest disease-remission duration was 2884 days. Five additional retrospective studies in mixed population of refractory primary or secondary AIHA tested the efficacy and toxicity of rituximab [23–27]. Either in children or in adults, overall response rate was up to 82%. About the side effects from the treatment, only one of the studies [23] showed 2 patients with severe infections and 1 patient with a myocardial infarction. The most severe potential long-term complication of rituximab treatment was reported by Carson et al. [28] with progressive multifocal leukoencephalopathy in 2 patients in 57 cases. Most recently, Barcellini et al. [29] completed a clinical trial to investigate the efficacy, safety, and response duration of low-dose rituximab (100 mg fixed dose for 4 weekly infusions) together with a short course of steroids as first- or second-line therapy in 23 patients with primary AIHA. They showed 82.6% overall response at month +2, to 90% at months +6 and +12, and the better response in WAIHA (100% overall response at all time points) than in cold AIHA (average, 60%). The treatment was well tolerated with no adverse events or infections; retreatment was also effective. Furthermore, rituximab has also been shown to be effective in common variable immunodeficiency- (CVID-) associated WAIHA. Gobert et al. performed the multicenter retrospective study to assess efficacy and safety of rituximab in patients with CVID-associated ITP/AHA. The overall initial response rate to rituximab reached 85% including 74% complete responses. After a mean follow-up of 39 ± 30 months after rituximab first administration, 10 of the initial responders relapsed and retreatment with rituximab was successful in 7/9. Severe infections occurred after treatment with rituximab in eight patients (24%); four of whom were not on immunoglobulin replacement therapy. They accordingly concluded that rituximab appears to be highly effective and relatively safe for the management of CVID-associated WAIHA [30]. For patients with SLE-associated WAIHA, treatment of SLE (Systemic Lupus Erythematosus) will guide the way to curative therapy. Since the main cause in SLE is the loss of B lymphocytes tolerance, targeting B lymphocytes with rituximab against the B-cell-specific calcium channel CD20 to deplete B cells from the body and lower the circulating autoantibody levels would be an effective method. There are different studies for refractory SLE-associated WAIHA with various results showing adequate safety and efficacy profiles [31–33]. In an Exploratory Phase II/III SLE Evaluation of Rituximab (EXPLORER), the only adequately powered, double-blind trial demonstrated that rituximab decreased B cells and anti-dsDNA autoantibody and increased C3 and C4 levels [34]. In a retrospective analysis of 53 patients with refractory AIHA treated with rituximab in Belgium, the overall response rate was 79% with 47% complete response and 32% of partial response rate [35]. Mild infusion reaction including hypotension and fever is the most common complication of rituximab, and incidence of serious infection is very low [36].

As mentioned above, there is no doubt that the benefit/risk ratio for rituximab is high. Nevertheless, rituximab is not licensed for WAIHA so far due to the lack of any randomized trials. As the evidence of rituximab treatments increases, this issue might be solved in the near future. After then, for WAIHA patients whether primary or secondary, rituximab could be considered as the first choice, thus offering a way to obtain remissions without the toxicities associated with corticosteroids and other immunosuppressive agents.

### 3.2. Immunotherapy by Other Monoclonal Antibodies

Monoclonal antibody represents a major advance toward a targeted therapy that can dramatically improve the treatment effect with a substantial reduction of toxicity derived from therapy. In addition to the successful use of rituximab in WAIHA, Robak reported a second-generation, fully human, anti-CD20 monoclonal antibody with enhanced Fc effector function based on an IgG1 kappa immunoglobulin framework [37]. This newer antibody interacts with a different epitope from rituximab, which is located in the smaller extracellular loop of CD20 [38], giving it a higher binding affinity. Due to these improved properties, it received accelerated approval from the U.S. FDA in October 2009 and was granted a conditional marketing authorization by the European Medicines Agency in April 2010 for the treatment of patients with chronic lymphocytic leukemia (CLL) refractory to fludarabine and alemtuzumab [39]. Currently, NCCN guidelines provide the recommendation for use of ofatumumab in previously treated CLL patients [40]. Preliminary data show an overall response rate of 77%, including a CR rate of 32% in the 500 mg dosing arm and a CR rate of 50% in the 1000 mg dosing arm [41]. Ofatumumab has also shown potential in treating B-cell non-Hodgkin's lymphoma [40]. Since CLL and B-cell non-Hodgkin's lymphoma are both the main underlying conditions of secondary WAIHA, eradicating these underlying causes by this newly developed mAb will guarantee the improvement of related WAIHA. As the clinical trial data are accumulating, its wide use in clinics will be in the range soon.

There is another recently developed mAb, Alemtuzumab, which might be used in treating WAIHA. Alemtuzumab is a fully humanized IgG1-type mAb directed against CD52 expressed on human B and T cells, natural killer cells, eosinophils, and macrophages [42, 43]. This antibody kills target cells by complement- and/or antibody-dependent cellular cytotoxicity, but seems also to be capable of inducing direct apoptosis via caspase-dependent and -independent mechanisms [44, 45]. Clinically, the antibody reduces normal lymphocytes of both B and T lineages, resulting in a profound and occasionally long-lasting lymphopenia with concomitant immunosuppression. The first use of this mAb in treating autoimmune hemolytic anemia was reported by Willis et al. [46]. They treated two patients with AIHA: one reached CR with red cell independence of corticosteroids, the other a PR. Recent documentations on successful use of this mAb are from Karlsson et al. [47], Laurenti et al. [48], and Royer et al. [49]. In Karlsson et al.'s study [47], 5 patients with advanced B-CLL-related severe transfusion-dependent AIHA received subcutaneous or intravenous alemtuzumab and showed good response to the treatment with the mean hemoglobin increased from 7.2 g/dL at the baseline to 11.9 g/dL at the end. Later on, Laurenti et al. did a research on the efficacy and safety of alemtuzumab treatment and confirmed that this monoclonal antibody is effective in the treatment of severe, refractory, and secondary AIHA [48]. Royer's data are consistent with the above two reports [49]. According to the reported data [50], conventional therapies for WAIHA, such as corticosteroids, splenectomy, and immunosuppressive agents, may not induce complete resolution in all patients, and relapses are common. Thus, the successful use of monoclonal antibodies such as rituximab and alemtuzumab for the management of WAIHA means new therapeutic thoughts to eradicate the underlying causes of secondary WAIHA or completely cure the idiopathic WAIHA.

## 4. Elucidation of Mechanism of WAIHA Contributes to Developing Novel Immunotherapy Strategies

Production of warm reactive autoantibodies, IgG, IgM, and IgA that target the body's own red blood cells (RBC) is the main cause of warm autoimmune hemolytic anemia (WAIHA) [5, 6]. The etiology underlying the pathogenesis of such autoantibodies is still unclear. Possible mechanisms that produce a breakdown of immunologic tolerance leading to WAIHA include the possible roles of RBC autoantigens and the complement systems, the lack of effective presentation of autoantigens, functional abnormalities of B and T cells resulting in polyclonal lymphocyte activation and alteration of cytokine production, and the role of immunoregulatory T cells [7]. For a secondary WAIHA, underlying causes may be associated with systemic lupus erythematosus, malignant tumors, benign ovarian teratomas, lymphoproliferative disorders such as chronic lymphocytic leukemia and non-Hodgkin lymphomas, or drugs. Understanding these mechanisms is vital for developing antigen-specific immunotherapies to treat the disease.

Autoimmune hemolytic anemia (AIHA) has no known predisposition. There is no familial hereditary component, no age preselection, and no identified genetic background making an individual susceptible to develop AIHA [51]. Direct antiglobulin test (DAT) through cytofluorometry and specific diagnostic monoclonal antibodies (mAbs) allow for a better understanding of AIHA triggers. Genotyping blood groups and narrowing down the blood type subspecificities with diagnostic mAbs help to define the triggering autoantigens. Obviously, these new technologies will play important roles in investigating the mechanism of WAIHA. As we know, IgM is usually involved in triggering AIHA with cold agglutinins; however, its role in warm AIHA was recently reported in a patient with primary Sjogren's syndrome [52]. In this case, authors detected the warm reactive IgM antibodies on the surface of red blood cells by cytofluorometry for the first time. Since then, the pathophysiology of IgM in human autoimmune disease has attracted much attention, which thus led to a study focus on the immunoglobulin isotypes in autoimmune hemolytic anemia. Such studies have not only guided the paradigm for treatment, but also extended the knowledge of the role of this molecule in the induction of AIHA [53]. Although considerable work is needed to delineate the exact role of IgM in warm AIHA and to explore its role as a key modulator of autoaggressive responses, the paradigm for treatment with immunotherapy approaches has formed on the basis of IgM pathophysiology.

Studies of animal models and human AIHA demonstrate that the activation of autoreactive helper T (Th) cells by antigen presenting cells (APCs) is a key event in the induction of disease [54]. Therefore, the roles of Th cells (Th1, Th2, and Th17) in human AIHA reviewed by Fagiolo [55] could provide new opportunities to treat WAIHA with immunotherapy. With synthetic peptides that are recognized by Th cells, the effector Th response can be suppressed [56]. Rhesus (Rh) proteins, the dominant human RBC autoantigens [57, 58], have also been shown to be able to activate Th cells specific for epitopes on Rh proteins in the patients with primary warm type AIHA [59]. The activation of these specific Th cells hence holds out the prospect of safe, effective treatments for WAIHA. The evidence presented by Hall et al. [60] and Xu et al. [61] indicated alternative ways to develop potential new immunotherapy methods by clarifying the roles of Th17 cells in the development of AIHA. Blocking the activity of the Th17 pathway may be a complementary or more effective treatment strategy than inhibiting Th1 responses. Alternatively, there are possibilities of reinducing tolerance to the specific autoantigenic peptides that drive pathogenic Th-cell responses. Epitopes capable of inducing Th17 cytokine can be included as targets in this type of immunotherapies for AIHA. Attributing to insights into these pathogenic mechanisms, more and more new treatments have emerged, especially through monoclonal antibodies directing against certain B- or T-cell type. A fully human CD32 mAb has recently entered into the preclinical study for treating immune hemolytic anemia [62]. Although the evidence on the clinical use of these newly developed strategies in treating WAIHA is still in paucity, increasing survival rates and decreasing levels of life-threatening infections in patients revealed great prospects of immunotherapy for WAIHA [63].

## 5. Conclusions

The first choice of treating WAIHA is steroids; however, a proportion of patients will not respond well to steroids. Although two-thirds of them respond to this treatment, they usually require maintenance therapy or even become refractory to drugs due to long-term therapy [64]. Alternative options include splenectomy and immunosuppressors. For the severe anemia and the patient for whom none of the known drugs have worked, the treatment of last resort might turn to the high dose of immunosuppressive drugs and stem-cell transplantation. Unfortunately, the results of these methods were disappointing and produce strongly harmful side effects [65]. Owing to the dramatic changes in the therapeutic regimens employed in autoimmune disease over the past few years, the situation is improving. With soluble receptors, monoclonal antibodies, and molecular mimetics enhancing or gradually replacing conventional immunosuppressive therapies, new immunotherapy treatments targeting defined pathways of the formation of WAIHA have become more efficacious and less toxic. Established data have demonstrated that rituximab is efficacious and effective in treatments of WAIHA [22–28]. Alemtuzumab and Ofatumumab also found a way recently for treating WAIHA patients [40, 41, 47–49]. Paying more attention to antigens on red blood cells targeted by autoantibodies would be an important factor to develop novel therapeutic mAbs. Exploring monoclonal antibodies against disease-related cytokines, such as IL-10, IL-12, and IL-17, can also be promising. Most importantly, the elucidation of immune mechanism of WAIHA is always the center of improving the quality of therapy for patients with WAIHA. Significant challenges in developing novel strategies of immunotherapy for WAIHA patients remain in the identification of optimal cellular targets, antibody forms, and treatment schedules for therapeutic applications.
